# Major Histocompatibility Complex I Mediates Immunological Tolerance of the Trophoblast during Pregnancy and May Mediate Rejection during Parturition

**DOI:** 10.1155/2014/579279

**Published:** 2014-04-09

**Authors:** Anna Rapacz-Leonard, Małgorzata Dąbrowska, Tomasz Janowski

**Affiliations:** Department of Animal Reproduction with Clinic, Faculty of Veterinary Medicine, University of Warmia and Mazury, ul. Oczapowskiego 14, 10-719 Olsztyn, Poland

## Abstract

During pregnancy in larger mammals, the maternal immune system must tolerate the fetus for months while resisting external infection. This tolerance is facilitated by immunological communication between the fetus and the mother, which is mediated by Major Histocompatibility Complex I (MHC I) proteins, by leukocytes, and by the cytokines secreted by the leukocytes. Fetal-maternal immunological communication also supports pregnancy by inducing physiological changes in the mother. If the mother “misunderstands” the signal sent by the fetus during pregnancy, the fetus will be miscarried or delivered preterm. Unlike any other maternal organ, the placenta can express paternal antigens. At parturition, paternal antigens are known to be expressed in cows and may be expressed in horses, possibly so that the maternal immune system will reject the placenta and help to expel it. This review compares fetal-maternal crosstalk that is mediated by the immune system in three species with pregnancies that last for nine months or longer: humans, cattle, and horses. It raises the possibility that immunological communication early in pregnancy may prepare the mother for successful expulsion of fetal membranes at parturition.

## 1. Introduction 

During pregnancy in larger mammals, the maternal immune system must tolerate the fetus for months. Although in mice tolerance is accomplished by suppression of maternal immune cells, species with longer pregnancies probably cannot suppress their immune systems to the same extent because this would make them prone to infections [1].

To examine how the immunological challenge of a long gestation period is met, we chose three well-studied species with pregnancies that last nine months or longer: humans, cattle, and horses. In these species, tolerance is mediated by Major Histocompatibility Complex proteins, by leukocytes, and by the cytokines secreted by the leukocytes [2]. If the mother “misunderstands” the signal sent by the fetus during pregnancy, the fetus will be miscarried or delivered preterm [3]. Interestingly, although it might be assumed that tolerance would be accomplished in all these species by “hiding” the fetus from the maternal immune system, paternally inherited antigens are expressed during early pregnancy by trophoblast cells in cattle and horses. At parturition, paternal antigens are known to be expressed in cows, possibly so that the maternal immune system will reject the placenta and help to expel it [4, 5].

This review compares fetal-maternal crosstalk that is mediated by the immune system in humans, cattle, and horses. It examines physiological pregnancy (in which gestation is not shortened and the fetus is not miscarried or delivered preterm), pathological pregnancy, and parturition. It suggests the hypothesis that, in horses and cows, the expression of paternal antigens by invading trophoblast cells may educate the maternal immune system and prepare it for rapid rejection of fetal membranes at parturition.

## 2. Major Histocompatibility Complex Class I (MHC I) 

### 2.1. MHC I Proteins Mediate Communication between the Fetus and the Mother

There are two classes of MHC I: classical and nonclassical (Table 1). Classical MHC are highly polymorphic, which means they have the ability to present many antigens including foreign antigens [6]. If cells express these foreign antigens they are attacked by cytotoxic T lymphocytes (CTL) [7].

Nonclassical MHC I are not polymorphic and present a so-called “zero” antigen. The “zero” antigen fills a groove in nonclassical MHC I proteins and is recognized by leukocytes as a maternal “self” antigen; however, it is not of maternal origin. The cells that express “zero” antigen are protected because cells that do not express any antigen are attacked by uterine Natural Killer cells (uNK) [8, 9].

Communication between MHC I and leukocytes (uterine Natural Killer cells, macrophages, and T lymphocytes) induces and maintains maternal tolerance during physiological pregnancy. In humans, cattle, and horses, expression of MHC I is increased by trophoblast cells that invade the endometrium as they become more exposed to the maternal immune system [1].

The pattern of expression of MHC I differs according to the species. In humans, the trophoblast expresses nonclassical MHC I. These nonclassical MHC I bind a “zero” antigen that protects the cells by binding with uNK [8, 9]. In cattle and horses, the invasive trophoblast expresses classical MHC I with paternal antigens [10, 11], and this pattern of expression stimulates a response from cytotoxic T lymphocytes (CTL) [6, 7]. The reasons for different patterns of expression of MHC I on invasive trophoblast are not clear, although this might be associated with the structure of the placenta in different species.

### 2.2. Humans (Invasive Placenta)

In humans, nonclassical MHC I enters the maternal circulation, which is probably facilitated by the invasive placenta structure (*hemochorial placenta*) that has been found in this species, as well as in apes, monkeys, and rodents [12]. In these species with invasive placentas, a part of the trophoblast that is called the extravillous trophoblast destroys 3 layers of endometrial tissue so that it can be in direct contact with maternal blood. The blood passes through a disk-shaped zone, which maternal arteries and veins access from the endometrium. Nourishment is passed to the fetus through 3 layers of cells in highly vascularized villi that sink into this disk and are washed by maternal blood [13]. To ensure that enough blood can circulate through this disk, blood pressure in the maternal arteries is increased by a process called spiral artery remodeling [8, 9, 14].

**Figure 1:**
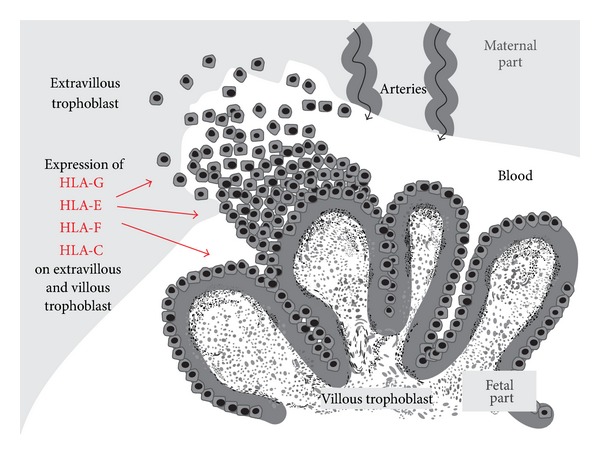
Expression of MHC I proteins (HLA) on the surface of the villous and extravillous trophoblast in humans. Only tolerance mediating HLA are expressed.

The structure and expression of the MHC I that mediate tolerance and support of pregnancy have been best defined in humans, in which MHC I are referred to as Human Leukocyte Antigen (HLA) (Table 1). Human classical MHC I are polymorphic and are known to exist in several classes [6]. Of these classes, only HLA-C bind “zero” antigens. HLA-C is expressed on the entire surface of the trophoblast villi (Figure 1) [15]. When HLA-C is bound by the KIR2D receptor on uterine Natural Killer cells (uNK), this leads to optimum blood supply to the trophoblast, thus supporting the fetus [16].

The nonclassical MHC I are not polymorphic, and “zero” antigens are expressed by all 3 known classes: HLA-E, HLA-F, and HLA-G [17–19]. These HLA are soluble and are expressed on the whole surface of the trophoblast villi (the villous and extravillous trophoblast) (Figure 1). Nonclassical HLA mediate both tolerance and support of pregnancy [1, 18–23].

Tolerance is induced by HLA-G, which is known to enter the maternal circulation and bind with the leukocytes immunoglobulin-like receptors (LIR-1 and LIR-2) on uNK, macrophages, and T lymphocytes. After binding, the leukocytes are inactivated and express more LIR-1 receptors [1, 16, 18–23] (Table 3).

Even in pathological pregnancies, only HLA that induce tolerance have been found, that is, HLA with “zero” antigens. Yang et al. [24] took samples from the trophoblast during pregnancy and cultured them with INF-gamma, a strong proinflammatory cytokine. Normally, when tissues are treated with this cytokine, they respond by expressing HLA-A and HLA-B. These are classical MHC I that induce inflammation and their expression of immune rejection might lead to recognition by cytotoxic T lymphocytes and immune rejection. However, the cultured trophoblast cells in this experiment continued to express only tolerance-inducing HLA-G. This suggests that the mechanisms that lead to expression of only HLA with “zero” antigen during pregnancy are extremely robust, although they remain unknown.

### 2.3. Cows (Noninvasive Placenta)

Ruminants are known to have noninvasive placentas [12]; of these species, cows have been studied the most. In noninvasive placentas, the trophoblast has no contact with maternal blood. Instead, nourishment is passed from the mother to the fetus through structures called placentomes [34]. In cows, there are 70–120 placentomes scattered throughout the entire placenta [35]. Placentomes consist of vascularized villi that originate in the trophoblast and the corresponding endometrial crypts into which the villi fit. Nourishment passes from the maternal to the fetal blood through six layers of cells, three in the endometrium and three in the trophoblast villi [12].

**Figure 2:**
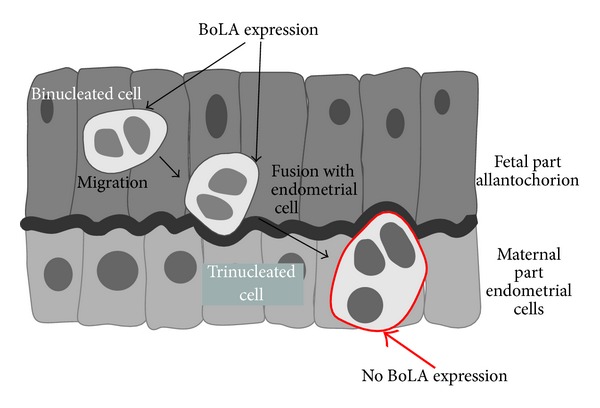
Expression of classical MHC I (BoLA) proteins in the cow placenta. Classical BoLA are expressed only on migrating binuclear cells and they disappear when binuclear cells fuse with endometrial cells (creating trinucleated cells).

In cows, MHC I are referred to as Bovine Leukocyte Antigen (BoLA) (Table 1). Unlike in humans, classical BoLA with paternal antigens are known to be expressed during physiological pregnancy, in addition to nonclassical BoLA with “zero” antigens [36–38]. Throughout all of pregnancy, the paternal antigens are expressed on binuclear cells, which have a role in supporting pregnancy. Binuclear cells originate in the trophoblast, although the exact details of their origin are unknown. The cells migrate from the trophoblast and invade the endometrium, where they fuse with endometrial cells to create giant trinuclear cells. These giant cells lose the paternal antigens and express no BoLA at all (Figure 2) [10, 36]. Giant cells help to stabilize pregnancy by secreting bovine placenta lactogen, which influences ovarian and placental steroidogenesis and alters maternal metabolism to support fetal growth and development [39].

In cows, nonclassical BoLA bind “zero” antigens, and they may have a role in inducing tolerance. Unlike humans, these nonclassical BoLA have not been found on the entire surface of the trophoblast but only on the regions between the placentomes (interplacentomal region) and between the villi (arcade region) [10]. Moreover, these BoLA have only been found during the last trimester of pregnancy, not throughout the entire pregnancy as in humans [2, 10]. Nonclassical BoLA are produced in both nonsoluble and soluble forms [37], so it can be speculated that the soluble BoLA also bind LIR-1 receptors on leukocytes in cows, which could inhibit the leukocytes, similar to as in humans.

During clone pregnancies in cows, classical BoLA with paternal antigens have been found on the trophoblast surface during the first month of pregnancy [40]. It is speculated that this presentation of paternal antigens is connected with the high number of clone pregnancies that are lost due to attack by activated cytotoxic T lymphocytes (CTL) [37, 40]. It is not known why paternal antigens are only presented on the trophoblast in these pregnancies, but it is likely that this is due to altered gene expression caused by the nuclear transfer process [40].

#### 2.3.1. Parturition in Cows

Although little is known about immunological activity at the time of parturition, research in cows suggests that expulsion of fetal membranes is promoted when the maternal immune system rejects paternal antigens that are presented by the fetal membranes [4, 5]. Classical BoLA with paternal antigens have been found to be expressed by cows at parturition [41–43]. This expression is probably necessary for placenta maturation. The trophoblast villi are the contact zone in the placentomes, and, up to one month before parturition, the endometrial epithelium undergoes a thinning process and then disappears completely. This histological change leads to loosening of the contact area, so that the trophoblast epithelium contacts the connective tissue of the endometrium (the placenta changes from* epitheliochorial* to* synepitheliochorial*) [44, 45].

In addition, when paternal antigens are presented by classical BoLA protein on the surface of chorion cells [46], the antigens are recognized by T lymphocytes (CD 8+). This recognition can be seen as an increased migration of these lymphocytes to the placenta surface. The increased chemotactic activity of the lymphocytes has been well investigated in cows and this activity decreases when cows retain fetal membranes [44].

In a study by Benedictus et al. [47], classical BoLA compatibility from the point of view of the immune systems of both the calf and the dam gave a significantly higher risk of retention of fetal membranes with an odds ratio of 16.25. In a study by Streyl et al. [48] that compared mRNA expression 6 to 26 days before parturition with expression during physiological parturition, upregulation of certain genes during parturition suggested that increased numbers of leukocytes were present in the fetal-maternal contact zones in the placentomes. Nothing further is known about the immunological mechanisms that lead to retention of fetal membranes in cows or in other species. However, it is possible that immunological communication during pregnancy may prepare the cow for rejection and expulsion of fetal membranes at parturition. This possibility is explored in Section 4.

### 2.4. Horses (“Semi-Invasive” Placenta)

**Figure 3:**
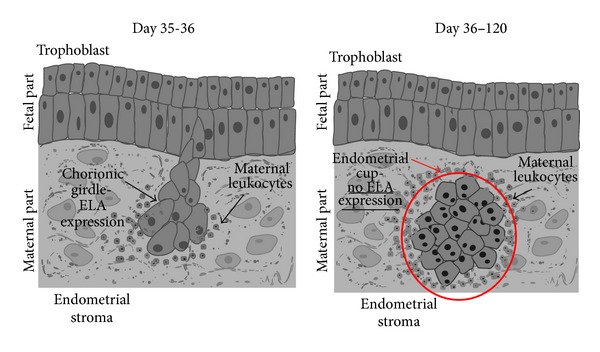
Expression of classical MHC I (ELA) proteins in the horse placenta. ELA are expressed only on the migrating chorionic girdle. Then ELA expression is downregulated when endometrial cups are created. After some time, the endometrial cups degenerate, and they have not been detected after day 120 of pregnancy.

Horses have an* epitheliochorial* placenta, as do species that are classified as having a noninvasive placenta [12]. However, because the horse placenta has a subpopulation of highly invasive trophoblast cells (called the chorionic girdle), the authors here will refer to this kind of placenta as “semi-invasive.” This subpopulation of invasive cells forms a chorionic girdle that encircles the fetus. By day 35 of pregnancy, cells of the chorionic girdle adhere to the endometrial epithelium and begin to invade the endometrium [49–52] (Figure 3). The aggressive invasive behavior of these cells is similar to the behavior of cells in the human extravillous trophoblast and to metastatic tumor cells [53]. The chorionic girdle disappears at about days 36–38 of pregnancy [50, 54, 55].

Chorionic girdle cells have been found to express MHC I, which is referred to in horses as Equine Leukocyte Antigen (ELA) [49, 56]. This expression quickly diminishes after invasion and is not found in mature endometrial cups [57, 58]. The expression of ELA by other cells during horse pregnancy and at any other time during pregnancy has not been investigated. Moreover, it has not been established whether nonclassical or classical ELA are expressed, nor whether the ELA bind “zero” or paternal antigens. Evidence for the binding of paternal antigens by what would probably be classical ELA is the fact that CD8+ T leukocytes have been found to be attracted to the cells that express ELA [29]. These CD8+ T leukocytes have been found around the chorionic girdle on the same days that MHC I was expressed [11, 30, 51, 56, 57, 59, 60]. Antibodies to paternal antigens that were produced by B lymphocytes have been found at stable levels in the peripheral blood throughout the rest of pregnancy [54, 61–63]. However, CD8+ T leukocytes were not found to attack trophoblast cells that were expressing ELA. This may be because the paternal antigens are expressed for too short a time for the immune system to prepare itself to attack the paternal-antigen presenting cells (the chorionic girdle disappears on days 36–38) [50, 51, 54, 55].

When the chorionic girdle invades the endometrium, it forms distinct nodules in the endometrial stroma. These nodules are called endometrial cups; mature endometrial cups do not express any ELA [57, 58] (Figure 3). Endometrial cups produce equine chorionic gonadotropin (eCG) [64, 65]. eCG stimulates the ovary to produce additional corpus lutea [66]. The corpus lutea secrete a high level of progesterone, which supports the pregnancy [3]. The endometrial cups remain and continue to secrete eCG until about days 90–120, by which time they have degenerated [54].

The chorionic girdle and the endometrial cups are important for maintaining pregnancy. In donkey-in-horse pregnancies there is no chorionic girdle or endometrial cups, and it has been speculated that this is related to the high rate of abortions in these pregnancies [67, 68].

Cells in the endometrial cups have IL-22R1 receptors which bind IL-22, which is secreted by the chorionic girdle. Binding of Il-22 helps to maintain mucosal immunity, by facilitating endometrial reepithelization and upregulating antimicrobial proteins [50].

## 3. Leukocytes 

### 3.1. Leukocytes (uNK, Macrophages, T Lymphocytes) Not Only Tolerate Pregnancy But Also Support It

During pregnancy in all three species described here, maternal leukocytes behave differently in the uterus than they do in the rest of the mother's body. When uNK from humans [9], macrophages from humans and cows [69, 70], and T lymphocytes from horses [30] have been taken from pregnant uteruses and compared to leukocytes taken from the peripheral blood, the uterine leukocytes were found to be inhibited from engaging in normal immune responses, although the mother is able to resist general infection. This phenomenon is known as split immune tolerance [30].

### 3.2. Uterine Natural Killer Cells (uNK)

*In Humans, Uterine Natural Killer Cells Support the Growth and Development of the Fetal Unit.* In humans, uNK cells are the most abundant leukocytes in the placenta (Table 2), and their number remains constant throughout pregnancy [31]. uNK and their receptors are a type of NK cells that are unique to the uterus and they differ structurally from peripheral NK [71, 72]. The phenotype of uNK (CD56^BRIGHT^, CD16^−^, and CD3^−^) distinguishes them from NK in peripheral blood (CD56^DIM^, CD16^BRIGHT^, and CD3^−^) [62]. uNK do not attack the trophoblast; this is mediated by nonclassical MHC I (HLA-G), as mentioned before (Section 2.2).

uNK cells change their structure as pregnancy progresses, and these changes are related to the roles that uNK play in inducing tolerance and support of the fetus and placenta. In the first trimester uNK are granulated [31]. The granules contain angiogenic growth factor and vascular endothelial factor C. Angiogenic growth factor is released when HLA-G binds to the uNK LIR-1 receptor [73]. This growth factor promotes spiral artery remodeling and may increase vascularization in the syncytial villi. Vascular endothelial factor C stimulates the trophoblast to produce TAP-1 protein. This protein induces HLA-G protein loading. This seems to be a feedback loop that helps to stabilize immunological tolerance of the fetus [74]. In the second trimester uNK undergo a degranulation process and in the third trimester only degranulated uNK cells are present in the endometrium [31]. When degranulation starts, uNK stop secreting the above factors and begin to secrete IFN-gamma. This cytokine inhibits the migration of trophoblast cells, protecting the uterus from too much destruction by these invasive cells [7, 31, 75]. Details about what induces uNK cells to change their structure have not been elucidated.

uNK also help support the fetus in other ways. They induce optimal blood supply for the fetus by participating in spiral artery remodeling [8, 9, 14, 16] when their KIR2D receptors bind HLA-C that is present on the surface of the trophoblast. uNK secrete the matrix metalloproteinases MMP2 and MMP9 during implantation on the 8th to 10th day after ovulation [76]. These enzymes break down fibrous proteins that are known as the extracellular matrix. By breaking down this matrix, these metalloproteinases reduce the intercellular gap between the trophoblast and the endometrium [77]. The mechanisms that induce uNK to secrete these metalloproteinases are unknown.

uNK are inhibited from attacking the fetal unit when their LIR-1 receptors bind soluble HLA-G that has entered the maternal circulation (as part of the extravillous trophoblast—Figure 1). This binding also causes the cells to express more inhibitory LIR-1 receptors. In addition, the number of inhibitory LIR-1 receptors increases both on uNK and on macrophages and T leukocytes when the KIR2DL4 receptors on uNK are bound by HLA-G [78–80]. It is not known if and how uNK communicate with the other leukocytes to effect this change in the number of their inhibitory receptors.

The functions of uNK are important for healthy pregnancy in humans. Altered numbers of uNK or decreased numbers of KIR2D receptors on uNK have been associated with fetal growth restriction and insufficient trophoblast invasion [16], miscarriage [81, 82], implantation failure [83], and preeclampsia [84].

So far, uNK have only been found in human pregnancies. Some NK cells have been found in the horse placenta [54], but none in cows; therefore all the information that we have about the activity of uNK comes from studies with humans.

### 3.3. Macrophages

*Macrophages Inhibit the Activity of Other Leukocytes.* Information about the activity of uterine macrophages also comes mostly from studies with humans. Macrophages protect pregnancy in humans throughout all of gestation by inhibiting the immune response by uNK, T lymphocytes, and other macrophages [70]. The LIR-1 receptor on macrophages is bound by HLA-G. After this happens, macrophages do not secrete the proinflammatory cytokines TNF-alpha and INF-gamma. Instead they synthesize more LIR-1 receptors and secrete prostaglandin PGE_2_, which suppresses the activity of other macrophages, T lymphocytes, and uNK [78–80].

CD9 protein is expressed by uterine macrophages; it is bound by pregnancy specific protein (PSG), which is secreted by the trophoblast. After these receptors are bound, the macrophages secrete IL-10, which inhibits secretion of TNF-alpha by uNK and T lymphocytes [85, 86].

To prevent activation of maternal T lymphocytes, macrophages reduce expression of the costimulatory molecules CD80 and CD86 and express indoleamine-2,3-dioxygenase (IDO) [78–80, 86]. IDO is an immunomodulatory enzyme that catalyses degradation of essential L-tryptophan, which inhibits proliferation of T lymphocytes and prevents their activation [87].

### 3.4. T Lymphocytes

#### 3.4.1. T Lymphocytes Tolerate the Fetal Unit, and They May Protect It and Support Its Development

*Humans.* For tolerance of pregnancy, there is a bias toward cytokines production by CD4+ T-helper (T_H_2) cells and against cytokines production by CD4+ T_H_1 cells [88]. Naïve CD4+ T lymphocytes recognize HLA-G “zero” antigens (presented on antigen presenting cells) when the antigens bind with their LIR-1 receptors. This recognition inhibits proliferation of CD4+ T-cells, induces their long-term unresponsiveness, and causes differentiation of the CD4+ T-cells into suppressive T_H_2 cells [89]. Also, secretion of proinflammatory TNF-alpha by CD4+ T_H_1 cells is inhibited by IL-10 secreted by macrophages [23].

By avoiding the T_H_1 response, the fetal unit avoids attack by activated CD8+ cells (CTL). CTL cells are also inhibited when their LIR-1 receptors bind with HLA-G. Moreover, suppressor CD4+ CD25 cells inhibit activation of CTL cells by the same IDO mechanism as macrophages (mentioned in the “Macrophages” section) [6]. However, CD8+ T lymphocytes are present at the site where placenta implantation takes place. These lymphocytes are thought to protect the pregnancy against external antigens and to support trophoblast growth by secreting IL-8, which promotes trophoblast invasion [31].

*Horses.* In mares, CD4+ and CD8+ T lymphocytes have been found to cluster around the cells of the invading chorionic girdle, and around the early, mature, and dying endometrial cups (which are formed by the fusion of the girdle cells with endometrial cells) in greater numbers than are found in the rest of the endometrium [29]. As detailed in the section on horse MHC I (ELA), the invading cells express ELA with paternal antigens, but the mature and dying cups do not, and it is unclear how T cells recognize these cells that do not express ELA [57, 58]. It is also unclear why CTL do not destroy the ELA-expressing cells of the invading chorionic girdle and of the early endometrial cup. It appears that both a systemic increase in T-cell tolerance during pregnancy and an unknown inhibitory factor that is produced by trophoblast cells help protect the ELA-expressing cells [55, 90–92].

## 4. Discussion

To mediate tolerance by the maternal immune system during pregnancies in humans, cows, and horses, which normally last for nine months or longer, MHC I present “zero” antigens, which are recognized by leukocytes. These leukocytes secrete cytokines that increase expression of “zero” antigens, further induce tolerance in other leukocytes, and support pregnancy. Although the details of this process are best known in humans, there are findings of immune activity in other species that have not yet been investigated in humans. Much remains to be done both to clarify the mechanisms of maternal immunological tolerance in individual species and to find out which mechanisms are common to all placental mammals and which species are specific. As Bainbridge [1] points out, the differences between immunological fetal-maternal crosstalk in humans, cattle, and horses may exist because their common ancestor may have had a short gestation period. Thus, these species may have independently evolved different mechanisms to protect the fetus from longer exposure to the maternal immune system.

However, it is interesting to note that, in all three species examined in this review, invasive trophoblast cells increase their expression of MHC I as they become more exposed to the maternal immune system. Bainbridge [1] has advanced three hypotheses to explain this phenomenon: (1) MHC on trophoblast cells may help them adhere to and invade maternal tissue, (2) MHC expression may protect the invading cell from the maternal immune system, although it is difficult to understand how the paternal antigens on cattle and horse cells would pacify the maternal immune system, and (3) this expression of MHC may protect the entire fetoplacental unit from the maternal immune system, at least in humans, where HLA-G has been found to be able to suppress the proliferation of peripheral blood lymphocytes [93].

**Figure 4:**
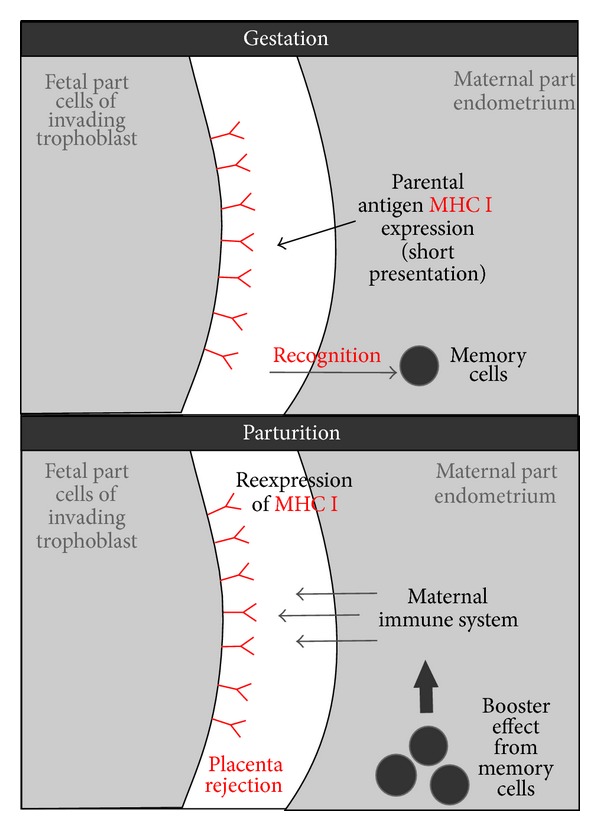
Hypothetical mechanism explaining why expression of classical MHC I during gestation is needed for fetal membranes rejection during parturition. In cows and horses trophoblast has no access to maternal blood (in contrast to humans). In these species trophoblast expresses classical MHC I with paternal antigens during pregnancy. This expression “teaches” maternal memory cells, which recognize paternal (foreign) antigen. If during parturition fetal membranes reexpress classical MHC I with paternal antigens they will stimulate booster effect from maternal memory cells and lead to fetal membranes rejection.

We suggest a fourth hypothesis be added to the three above: in horses and cows, the expression of paternal antigens by invading trophoblast cells may educate the maternal immune system and prepare it for rapid rejection of fetal membranes at parturition (Figure 4). In horses, when trophoblast cells that display paternal antigens invade the endometrium, CD8+ T lymphocytes are attracted to those cells [11, 30, 51, 56, 57, 59]. After this, memory CD8+ T-cells may persist in the mare. This persistence could prepare the immune system for a rapid response to paternal antigens that are presented at or just before parturition, if the unknown factor that inhibited the T-cells is not present in the uterus at parturition. After invasion by chorionic girdle cells, antibodies to paternal antigens that were produced by B lymphocytes have been found to remain at stable levels in the mare's peripheral blood throughout the rest of pregnancy [52, 61–63]. At parturition, the mare's immune response to paternal antigens may be similar to what is known to occur in humans when macrophages recognize foreign antigens: the macrophages secrete proinflammatory TNF-alpha and INF-gamma, which activate both other macrophages and T lymphocytes [85]. Similar mechanisms in cows would help explain why classical MHC I compatibility from the point of view of the immune systems of both the calf and the dam gave a significantly higher risk of retention of fetal membranes [47].

## 5. Conclusion

In mammalian species in which pregnancy lasts for months, the maternal immune system must be able to resist infection while tolerating paternal antigens that are expressed by the fetus. Because of the length of pregnancy in these species, simply relying on extensive suppression of the mother's immune response is probably too risky. Humans, cows, and horses all have gestation periods of nine months or longer, and they have evolved similar mechanisms for meeting this immunological challenge. When MHC I present “zero” antigens, these antigens are recognized by leukocytes, and these leukocytes secrete cytokines which induce tolerance in other leukocytes, stimulate the expression of more “zero” antigens, and help support pregnancy. The details of this process differ between the three species, and much needs to be done to determine which mechanisms are common to all three species, and which are different. In addition, there are reports that suggest that immunological communication may prepare for and promote rapid rejection of fetal membranes during parturition in cows and horses, but whether or how this is done also needs to be determined.

## Figures and Tables

**Table 1:** Classes of MHC I expressed in humans, cows, and horses [6, 25–28].

MHC I	Humans	Cows	Horses
Classical	HLA-A HLA-B HLA-C	BoLA-A10, BoLA-A11, BoLA-A13, BoLA-A14, BoLA-A15, BoLA-A17, BoLA-A19, BoLA-A20, BoLA-CA24, BoLA-CA42B, BoLA-CC1, BoLA-w12.1, BoLA-A12 (w12B), BoLA-w7, BoLA-w9.2	ELA-A1, ELA-A2, ELA-A3, ELA-A4, ELA-A5, ELA-A6, ELA-A7, ELA-A8, ELA-A9, ELA-A10, ELA-A14, ELA-A15, ELA-A19, ELA-W11, ELA-W13

Nonclassical	HLA-E HLA-F HLA-G	BoLA-NC1 BoLA-NC2 BoLA-NC3 BoLA-NC4 MICA MICB	ELA-A1* ELA-C1* ELA-E1*

*From [25].

**Table 2:** Leukocytes that have been found in the placenta during physiological pregnancy in humans, cows, and horses [29–33].

	Humans	Cows	Horses
Leukocytes in placenta	Three major populations: (i) **Uterine Natural Killer (uNK) cells ** (ii) **Macrophages** (iii) **T lymphocytes** Other less abundant leukocyte populations: (i) Dendritic cells (ii) Natural Killer T (NKT) (iii) Regulatory T cells	**Endometrial macrophages** (as much as half of all immune cells) Other less abundant leukocyte populations: (i) Dendritic cells (ii) T lymphocytes	Mostly **T lymphocytes** Other less abundant leukocyte populations: (i) B lymphocytes (ii) NK cells (iii) Eosinophils

**Table 3:** Definitions of receptors and molecules mentioned in the paper.

Name	Full name	Presenting cell	Function	Synonyms
KIR2D	Killer cell immunoglobulin-like receptor	NK cells	Specific for HLA-C; inhibitory effect on NK cell	CD158 and 2DL3

KIR2DL4	Killer cell immunoglobulin-like receptor	NK cells	Binds MHC I and activates NK cells	CD158D, KIR103, 2DL4, and KIR103AS

LIR-1	Leukocytes immunoglobulin-like receptor 1	Immune cells	Binds MHC I and inhibits stimulation	CD85, ILT2, MIR7, LILRB1, and Ig-like receptor 1

LIR-2	Leukocytes immunoglobulin-like receptor 2	Immune cells	Binds MHC I and inhibits stimulation	CD58D, ILT4, MIR-10, 2

CD80	CD80 molecule	Macrophages and activated B lymphocytes	Involved in costimulatory signal for T-lymphocytes activation and works together with CD86	CD28LG, CD28LG1, and LAB7

CD86	CD86 molecule	Antigen presenting cells	Involved in costimulatory signal for T-lymphocytes activation and works together with CD80	CD28LG2, LAB72, B7-2, and B70

CD9	CD9 molecule	Cells exosomes	Mediates signal transduction events for regulation of cell development, activation, growth, and motility	MIC3, BTCC-1, DRAP-27, TSPAN-29, and MRP-1
