# Erratum to “Major Histocompatibility Complex I Mediates Immunological Tolerance of the Trophoblast during Pregnancy and May Mediate Rejection during Parturition”

**DOI:** 10.1155/2014/821530

**Published:** 2014-07-23

**Authors:** Anna Rapacz-Leonard, Małgorzata Dąbrowska, Tomasz Janowski

**Affiliations:** Department of Animal Reproduction with Clinic, Faculty of Veterinary Medicine, University of Warmia and Mazury, Ulica Oczapowskiego 14, 10-719 Olsztyn, Poland

In the paper, two inaccuracies have been found.

The first inaccuracy is in Section 2.2 on page 3 (in the pdf) in the following sentence: “These are classical MHC I that induce inflammation and their expression* of immune rejection* might lead to recognition by cytotoxic T lymphocytes and immune rejection.”

It should be without “of immune rejection.”

The second inaccuracy is in Section 3.4.1 on page 7 in the following sentence: “These lymphocytes are thought to protect the pregnancy against* external antigens* and to support trophoblast growth by secreting IL-8, which promotes trophoblast invasion.”

It is not “external antigens” but “pathogens.”

We apologize for this oversight and for any confusion that it has caused.

